# Diffusion-weighted Imaging Distortion in Prostate MRI: A Cross-sectional Study Comparing Supine and Prone Positioning

**DOI:** 10.1097/RLI.0000000000001245

**Published:** 2025-11-06

**Authors:** Kang-Lung Lee, Andrew B. Gill, Dimitri A. Kessler, Po-Hsiang Liao, Wellington Chishaya, Christopher Shepherd, Chao-Yu Guo, Iztok Caglic, Tristan Barrett

**Affiliations:** Department of Radiology, University of Cambridge, UK (K.L.L., A.B.G., D.A.K., T.B.); Department of Radiology, Cambridge University Hospitals NHS Foundation Trust, Addenbrooke’s Hospital, Cambridge, UK (K.L.L., W.C., C.S., I.C., T.B.); Department of Radiology, Taipei Veterans General Hospital, (K.L.L.); School of Medicine, National Yang Ming Chiao Tung University, (K.L.L., P.H.L.); Department of Emergency Medicine, Taipei Veterans General Hospital, Taipei, (P.H.L.); Institute of Statistics, National Yang Ming Chiao Tung University, Hsinchu, (P.H.L., C.Y.G.); Division of Biostatistics and Data Science, Institute of Public Health, National Yang Ming Chiao Tung University, Taipei, Taiwan (C.Y.G.); Division of Medicine, Centre for Medical Imaging, University College London, London, UK (I.C.)

**Keywords:** prostate, cancer, MRI, diffusion-weighted imaging, image distortion, patient positioning

## Abstract

**Objectives:**

Diffusion weighted imaging (DWI) is a key component of multiparametric (mp) prostate MRI. DWI using echo-planar techniques is susceptible to distortion at the recto-prostatic air-tissue interface. This study was to determine whether prone patient positioning reduces adjacent rectal air and DW image distortion when compared with standard-of-care supine positioning.

**Materials and Methods:**

This prospective study included consecutive patients undergoing mpMRI for suspected PCa between 2023 and 2024. Prostate segmentation was performed on DW and contrast-enhanced images. DWI distortion was measured quantitatively. Qualitative image quality of DWI and T2-weighted imaging (T2WI) was evaluated using PI-QUAL version 2; a separate 5-point clinically based Likert scale was employed to evaluate the volume of rectal air adjacent to the prostate.

**Results:**

Fifty-two patients were enrolled. In total, 58% of patients expressed a preference for supine imaging versus 20% for prone imaging. Qualitative DWI image quality improved significantly in the prone position [median: 4 (3 to 4)] versus supine [3 (1 to 4)]; *P* < 0.001. In contrast, prone T2WI quality [1 (1 to 1)] was significantly inferior than supine T2WI [3 (3-4)]; *P* < 0.001. Quantitative measures of rectal air were significantly lower for prone [1.13 cm^3^ (0.34-2.43)] compared with supine imaging [1.96 cm^3^ (0.47 to 5.81); *P* = 0.005]. There was no significant difference in distortion between prone [3.21 mm (2.42 to 3.82) and supine [2.95 mm (2.25 to 4.21)] positioning across all patients (*P* = 0.80); however, in patients with >4 cm^3^ of supine rectal air (n = 19), distortion was significantly reduced by prone imaging [3.49 mm (2.84 to 4.03)] compared with supine [4.60 mm (3.17 to 5.95)]; *P* = 0.02. The mean additional scanning time for the necessary prone imaging was 8 minutes 18 seconds.

**Conclusions:**

Prone positioning significantly reduces DWI distortion artefact when rectal air is present, but consistently results in degraded T2WI quality.

Prostate cancer (PCa) is the second leading cause of cancer-related mortality among men worldwide.^1^ Magnetic resonance imaging (MRI) is now established as a vital tool for the diagnosis, staging, and monitoring of PCa, with the Prostate Imaging Reporting and Data System (PI-RADS) v2.1 guidelines serving as a widely accepted framework for MRI acquisition and interpretation.^2^ Diffusion-weighted imaging (DWI) and T2-weighted imaging (T2WI) represent the dominant sequences in the peripheral (PZ) and transition (TZ) zones, respectively, playing central roles in diagnostic MRI evaluation.^3^

Delivering high-quality MRI images is crucial for accurate MRI-based assessment; however, image quality can be significantly affected by various technical and patient-related factors.^4^ Standard-of-care DWI is typically acquired using single-shot echo-planar imaging (EPI) techniques, which are highly susceptible to magnetic field inhomogeneities. One primary patient-related factor impacting DWI is the presence of rectal gas, which can disrupt magnetic field uniformity and cause susceptibility artefacts, especially near an air-tissue interface.^5,6^ Due to the anatomy and supine patient position during MRI scanning, gas rises anteriorly within the rectum, accumulating at the border of the posterior prostate and anterior rectal wall.^7^ Appreciable rectal gas accumulation is not uncommon, occurring in more than a third of patients.^7^

Recognizing this challenge, some authors have proposed scanning patients in a prone position as a potential method to minimize susceptibility artefacts around the prostate, improving image quality.^4,5,8^ However, to our knowledge, no studies have comprehensively investigated the effect of this approach on DWI quality at prostate MRI. Therefore, the purpose of this study was to compare the quantitative and qualitative image quality of prostate MRI acquired in both supine and prone positions. Specifically, we aimed to determine whether patients with a significant amount of rectal air in supine positions can be imaged prone to reduce artefacts and enhance DW image quality. In addition, the secondary aim was to evaluate the effect of prone positions on the image quality of T2WI.

## METHODS

### Recruitment

This prospective study with a retrospective analysis was conducted at a single university hospital between June 2023 and August 2024 and approved by the institutional review board (23/WS/0047), with all patients providing written informed consent. Inclusion criteria included treatment-naive men referred for prostate MRI due to suspected prostate cancer. Only those subjects who completed the entire scanning protocol were included in the analysis. Following the scan, participants were invited to complete a questionnaire to assess their comfort level comparing supine and prone positions. A 5-point scale was used to assess comfort levels, ranging from 1, indicating high comfort in the supine position, to 5, indicating high comfort in the prone position, and 3 indicating no preference.

### Image Acquisition

All imaging was performed on a 3.0T SIGNA Premier MR system (GE Healthcare, Waukesha, WI). To reduce bowel motion, patients were administered hyoscine butylbromide (Buscopan, 20 mg/mL, Boehringer, Germany) intravenously, unless clinically contraindicated (n = 12). As per routine local practice, patients were advised to evacuate the rectum before their MRI appointment; however, no further patient preparation techniques were employed. Patients were initially positioned supine in the magnet bore and scanned with axial T1-weighted imaging (T1WI) of the pelvis, T2WI of the prostate in axial and sagittal planes, axial DWI with corresponding ADC maps calculated on the scanner, and axial dynamic contrast-enhanced (DCE) MRI following gadobutrol (Gadovist, Bayer HealthCare) administration. Patients were then repositioned prone, and the same imaging sequences were repeated, except the axial T1WI and sagittal T2WI. For prone imaging, a shortened DCE series consisting of only 4 dynamic phases was acquired instead of the full sequence, without repeat contrast injection. Axial T2WI, axial ADC on both positions, the final phase of the supine DCE images (sDCE), and the first phase of the prone DCE images (pDCE) were extracted for subsequent segmentation and analysis. A detailed overview of the imaging protocol is provided in Table 1. In addition, the echo train length of the axial T2WI sequence was 16; for the axial DWI sequence, parallel imaging (ASSET) was used with an acceleration factor of 2, the echo spacing was 0.88 ms, the acquisition bandwidth was 111 kHz, and the field of view was square.

**TABLE 1:** Summary Table of Sequence Parameters for Axial T2-weighted Imaging (T2WI), Axial Diffusion-weighted Imaging (DWI), and Axial Dynamic Contrast-enhanced (DCE) MRI

	T2WI	DWI	DCE
Scanning sequence	SE	EP/SE	GR
MR acquisition type	2D	2D	3D
Repetition time (ms)	2401-3792	3775-4803	4.07
Echo time (ms)	96.6-164.3	66.2-66.7	1.75
Flip angle (°)	111	90	13
Fat-saturated	No	Yes	Yes
Diffusion b-values (s/mm²)	NA	100|750|1400	NA
No. averages	1.5	1|3|3	0.7
‍Temporal resolution (s)	NA	NA	8
Reconstructed matrix	512 × 512	256 × 256	256 × 256
In-plane voxel size (mm)	0.35 × 0.35	1.09 ×1.09	0.94 × 0.94
Slice thickness (mm)	3.0	3.0	3.0
Slice gap (mm)	0.0	0.0	0.0
Field of view (cm^2^)	18	28	24

SE indicates spin-echo; EP, echo-planar; GR, gradient-echo.

### Image Analysis

#### Qualitative Analysis of Image Quality

Two experienced uroradiologists (Authors T.B. and I.C.) with 15 and 10 years’ experience in prostate MRI reporting and considered experts (based on the number of mpMRIs interpreted^9,10^), assessed the quality of prostate mpMRI using the Prostate Imaging Quality (PI-QUAL v2) scoring system.^11^ PI-QUAL version 2 evaluates image quality using 10 specific criteria, comprising 4 for T2WI, 4 for DWI, and 2 for DCE.^11^ All images were evaluated on a Picture Archiving and Communication System (PACS) system. Throughout the assessment process, the radiologist remained blinded to patient clinical history, laboratory results, pathology findings, and existing radiology reports. In addition, the degree of rectal air distention was assessed visually using a previously described clinically relevant 5-point Likert scale, wherein scores of ≥4 are considered to represent clinically significant air. The scale was evaluated as followed: 1, rectal walls opposed; 2, minimal rectal air; 3, < 30% contact of rectal air on prostate with the posterior prostatic outline convex; 4, 30% to 80% contact of rectal air on prostate with the posterior prostatic outline flattened; 5, >80% contact of rectal air on prostate with posterior prostatic outline concave.^12^

### Quantitative Analysis of Image Distortion

**FIGURE 1:**
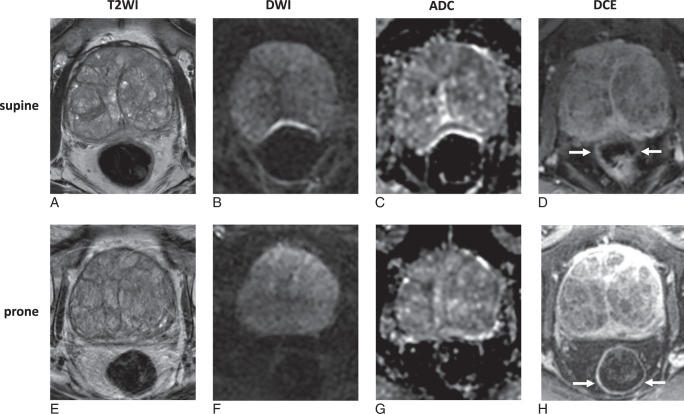
Prostate MR images from the same patient demonstrating improved image quality of prone DWI (F) and ADC map (G) compared with supine DWI (B) and ADC map (C). However, supine T2WI (A) showed superior image quality to prone T2WI, which demonstrates motion artefact (E). DCE sequence better demonstrates the anterior location of air at the rectoprostatic interface (arrows) on supine imaging (D) and the more posterior location (arrows) on supine imaging (H). ADC indicates apparent diffusion coefficient; DCE, dynamic contrast-enhanced T1-weighted imaging; DWI, diffusion-weighted imaging; T2WI, T2-weighted imaging.

An uroradiologist with 4 years’ experience in reading prostate MRI (Author K-L.L.) manually segmented the whole prostate gland on each slice of the ADC images and reference DCE images for all patients on both the supine and prone acquisitions using Stradview 7.2 (Cambridge University, Cambridge, UK, https://mi.eng.cam.ac.uk/Main/StradView). The reference images used were the final phase of sDCE and the first phase of pDCE since DCE can differentiate fecal materials from gas in the bowel (Fig. 1). T2W images were used for anatomic reference throughout the contouring process as needed. The outlining coverage from apex to base was identical for both supine and prone image sets. In addition, the rectum was outlined in the slices where the prostate was contoured. Rectal air was segmented using the Stradview semiautomated contouring function with a predefined signal intensity threshold to identify air (Supplemental Figure S1, Supplemental Digital Content 1, http://links.lww.com/RLI/B67).

A previously described algorithm was applied using in-house MATLAB code (Mathworks, Natick, MA), subject to validation and quality control testing described in (Ref. 13d), to calculate a quantitative measure of average distortion between the prostate outlines drawn on the ADC images and the reference outlines.^13^ In brief, the analysis pipeline for this process comprised the following steps: (1) production of volume masks from outlines on ADC and DCE reference images; (2) automatic co-registration of these volume masks; (3) assessment of the radial distortion within each imaged slice, as measured from the reference outline to the ADC outline, at 1 degree increments in polar coordinates; (4) calculation of the root-mean-square (RMS) of this measure over all imaged slices; and (5) calculation of the RMS distortion in the posterior half of the prostate organ. The values returned by the last 2 steps then supplied a quantitative measure (in mm) of the average overall degree of distortion in the ADC/DWI image volume for the whole prostate gland and its posterior half, respectively (Distortion_post_). Presence of rectal air within 20 mm of the posterior prostate was selected a priori based on clinical judgment as a proximity liable to result in susceptibility artefact on DWI. Automated MATLAB scripts programmed in-house were used to calculate the volume (in cm^3^) of rectal air within a 20 mm distance (Rectal Air_prox_) of the outlined prostate volume. This was computed by first evaluating the number of voxels in the intersection of the outlined prostate volume and all the outlined air pocket volumes and then multiplying this value by the volume of each voxel (Supplemental Figure S2, Supplemental Digital Content 1, http://links.lww.com/RLI/B67).

#### PI-RADS Categorization and Diagnostic Confidence

In addition to qualitatively assessing image quality, the senior radiologist retrospectively assigned a PI-RADS category^2^ and diagnostic confidence to four sequence combinations:

1. Supine T2 + Supine DWI + Supine DCE (standard-of-care protocol)
2. Supine T2 + Prone DWI + Supine DCE
3. Prone T2 + Prone DWI + Supine DCE
4. Prone T2 + Supine DWI + Supine DCE

For diagnostic confidence, the radiologist assigned a 1 to 5 Likert score for their confidence in making an assessment, as previously described,^14^ ranging from “very unsure” (score 1) to “very sure” (score 5).

### Statistical Analysis

The Shapiro-Wilk test suggested that variables did not follow a normal distribution. Descriptive statistics present median and interquartile ranges (IQR). The inter-reader agreement was evaluated using Gwet’s AC2 statistic with quadratic weighting. The interpretation of Gwet’s AC2 statistic was as follows: <0.00 (poor), 0.00 to 0.20 (slight), 0.21 to 0.40 (fair), 0.41 to 0.60 (moderate), 0.61 to 0.80 (substantial), and 0.81 to 1.00 (almost perfect).^15^ The analysis of PI-QUAL scores was based on the senior reader’s assessments since both readers showed similar categorization trends with almost perfect agreement based on Gwet’s AC2 statistic.

Qualitative assessment of rectal air was defined as appreciable for scores ≥4.^12^ A Fisher exact test was used to determine whether there was a significant difference in the dichotomized rectal air volume scores (score = 4 to 5 vs. 1 to 3) between the supine and prone positions.

Mean DWI distortion values (in mm) for the supine and prone imaging cases were then compared using the Wilcoxon signed-rank test for paired samples. To evaluate a possible threshold effect, ie, that distortion measures for supine and prone might diverge above a threshold rectal air volume, logistic regression examined the optimal threshold that dichotomized the volume of air within 20 mm of the prostate in the supine position, and the qualitative assessment of appreciable rectal air volume in the DCE images (Likert scale ≥4). This process yielded a threshold for the air volume [V_thresh-air_ (cm^3^)] likely to give rise to “significant distortion” in the images when the patient was supine. Mean distortion values were then compared for supine and prone positions in this subset of patients, who recorded supine rectal air >V_thres-air_ within 20 mm of the prostate organ volume. Logistic regression was performed using Python (version 3.13), and all other statistical tests were performed using in-house code written in MATLAB and R (version 4.2.2). *P* < 0.05 was considered statistically significant for all analyses.

## RESULTS

Fifty-three patients were scanned, with 52 included for analysis; one patient was excluded due to their inability to complete the scanning protocol (Table 2). The median patient age was 69 years (IQR: 64.8 to 72.5 y), and the median PSA level 4.68 ng/mL (IQR: 3.48 to 7.66 ng/mL). Of the 52 patients, 45 (87%) also completed the study questionnaire. Among these respondents, 26 (58%) preferred the supine position, 9 (20%) preferred the prone position, and 10 (22%) reported no difference in comfort between the 2 positions (Table 2). The mean total scanning time in the prone position for localizer and DWI sequences was 4 minutes 43 seconds (±33 seconds), with an additional 3 minutes 35 seconds (±1 min 1 s) needed for patient repositioning, resulting in an average total added scan time of 8 minutes 18 seconds.

**TABLE 2:** Characteristics of the Study Participants

Subject Details and Parameters	Values
Age	69 y (64.8-72.5)
PSA	4.68 ng/mL (3.48-7.66)
PI-RADS, n (%)	
1 or 2	31 (60)
3	4 (8)
4	9 (17)
5	8 (15)
Self-reported comfort levels, n (%)	
1 (highly comfortable with scanning in the supine position)	12 (27)
2 (somewhat comfortable with scanning in the supine position)	14 (31)
3 (no difference)	10 (22)
4 (somewhat comfortable with scanning in the prone position)	6 (13)
5 (highly comfortable with scanning in the prone position)	3 (7)

### Qualitative Analysis

**FIGURE 2:**
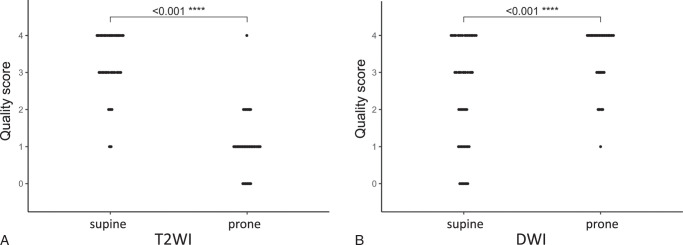
PI-QUAL v2 scores comparing supine and prone positions (A) T2-weighted imaging (T2WI) and (B) diffusion-weighted imaging (DWI). Supine T2WI quality scores were significantly higher than prone T2WI scores, whereas prone DWI quality scores were significantly higher than supine DWI scores. DWI indicates diffusion-weighted imaging; PI-QUAL, rostate imaging quality; T2WI, T2-weighted imaging.

**FIGURE 3:**
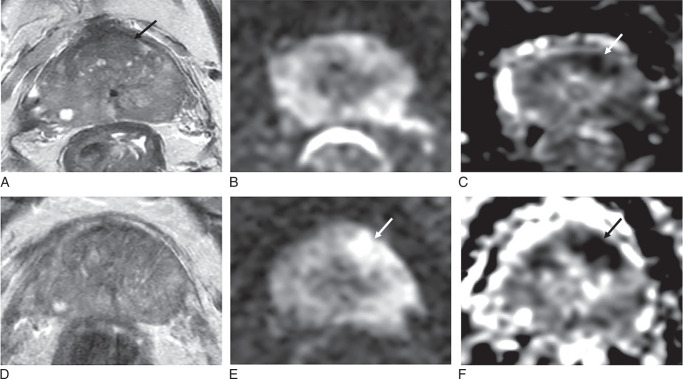
Differential scoring prone versus supine imaging. Seventy-one-year-old patient, PSA 7.31 ng/mL. A–C, Supine imaging shows 13 mm area of homogeneous low T2 signal in the left mid-anterior TZ with partial encapsulation, PI-RADS 2 (arrow, A), DWI, PI-QUAL 2/4 (B, C), shows marked low signal on ADC (arrow, C), but no significant change on b-1400 DWI (B); overall PI-RADS 3. DCE imaging in the TZ was negative (not shown). D–F, Prone T2 is nondiagnostic, PI-QUAL 0/4 (D), DWI shows 28 mm area of marked restricted diffusion (arrows) on b-1400 DWI (E) and ADC map (F), PI-RADS score 5. Overall, PI-RADS score 3 (2+1) using supine T2 and prone DWI, PI-RADS score 5 using prone imaging alone. Targeted biopsy shows Gleason 3+3=6 in 3/3 cores with 13 mm max tumor length.

**FIGURE 4:**
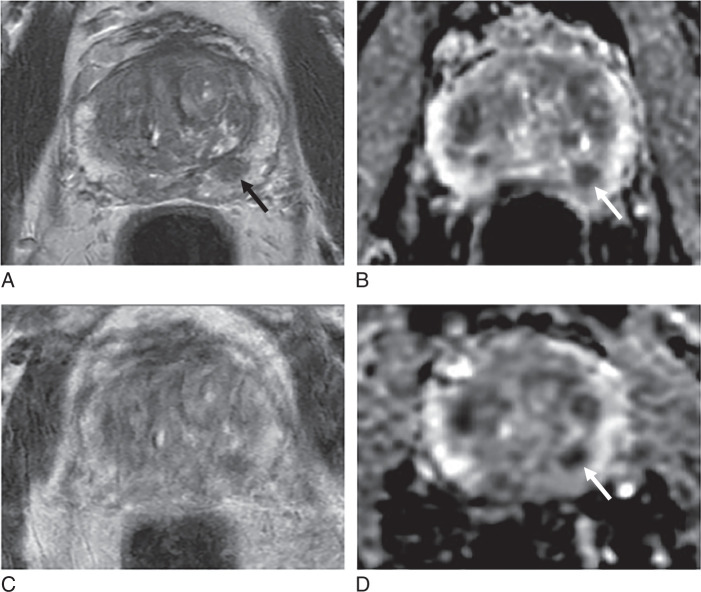
Example of nondiagnostic prone T2w images. Seventy-seven-year-old patient, PSA 4.67 ng/mL. A and B, Supine imaging shows a 9 mm area of focal low T2 signal in the left mid PZ (arrow, A) with restricted diffusion on ADC maps (arrow, B), PI-RADS 4. C and D, Prone T2 is nondiagnostic, PI-QUAL 0/4 (C), ADC map again shows a 9 mm area of marked restricted diffusion (arrow in D). Score is determined by the dominant PZ sequence of DWI and is overall PI-RADS 4 on prone and supine imaging. Targeted biopsy shows Gleason 3+4=7 in 1/3 cores with 6 mm max tumor length.

**FIGURE 5:**
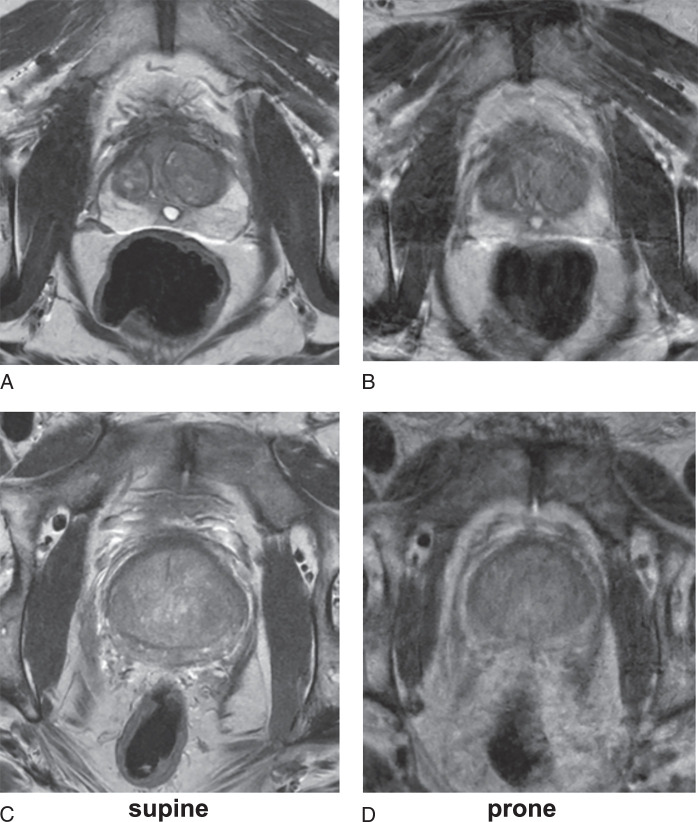
Prostate MR images from 2 different patients demonstrating inferior image quality of prone T2-weighted imaging due to motion artefact (B, D) compared with supine T2WI (A, C). PI-QUAL scores for T2 axial components: (A) 3/3, (B) 1/3 with a point scored for adequate signal-to-noise ratio, (C) 3/3, (D) 0/3.

The inter-reader agreement between the 2 radiologists on individual sequences was almost perfect, with Gwet’s AC2 values ranging from 0.87 to 0.94 (Supplemental Table S1, Supplemental Digital Content 2, http://links.lww.com/RLI/B68). DWI image quality, as assessed by PI-QUAL v2 with a 4-point scale according to the evaluation of the senior radiologist, significantly improved when patients were scanned prone (median: 4, IQR: 3-4) compared with supine (median: 3, IQR: 1-4; *P* < 0.001) (Figs. 1, 2A, 3, 4, Supplemental Table S2, Supplemental Digital Content 2, http://links.lww.com/RLI/B68). Conversely, T2WI quality scores, as assessed by PI-QUAL v2 with a 4-point scale, were significantly lower for prone images (median: 1, IQR: 1 to 1) compared with supine images (median: 3, IQR: 3 to 4; *P*<0.001) (Fig. 1, 2B, 3, 4, 5, Supplemental Table S2, Supplemental Digital Content 2, http://links.lww.com/RLI/B68). In the subset of 12 patients not receiving hyoscine butylbromide, the T2WI quality score remained significantly lower for prone (median: 1, IQR: 0.75 to 1) compared with supine imaging (median: 3, IQR: 2 to 4, *P* = 0.003). Appreciable rectal air (score ≥4) was observed more frequently in 20 (38%) supine scans compared with 12 (23%) prone scans, but the difference was not statistically significant (*P* = 0.07).

### Quantitative Analysis

**FIGURE 6:**
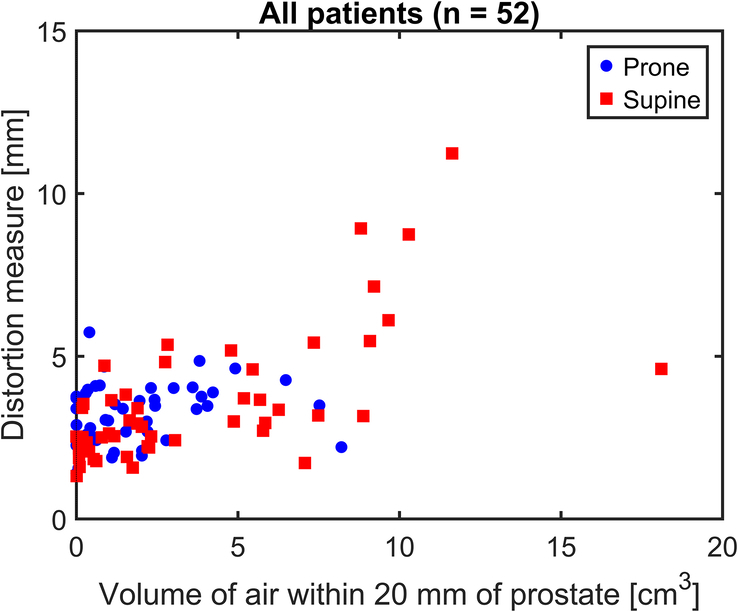
Scatter-plot of quantitative DWI distortion measure and volume of rectal air within 20 mm of the prostate. Values shown for patient positioning as prone (blue circles) and supine (red squares). It can be seen that there is little difference in the distribution of distortion values when the volume of rectal air is less than approximately 4 cm^3^. Above this threshold, image distortion increases relative to the volume of air and is predominantly seen with supine positioning.

**FIGURE 7:**
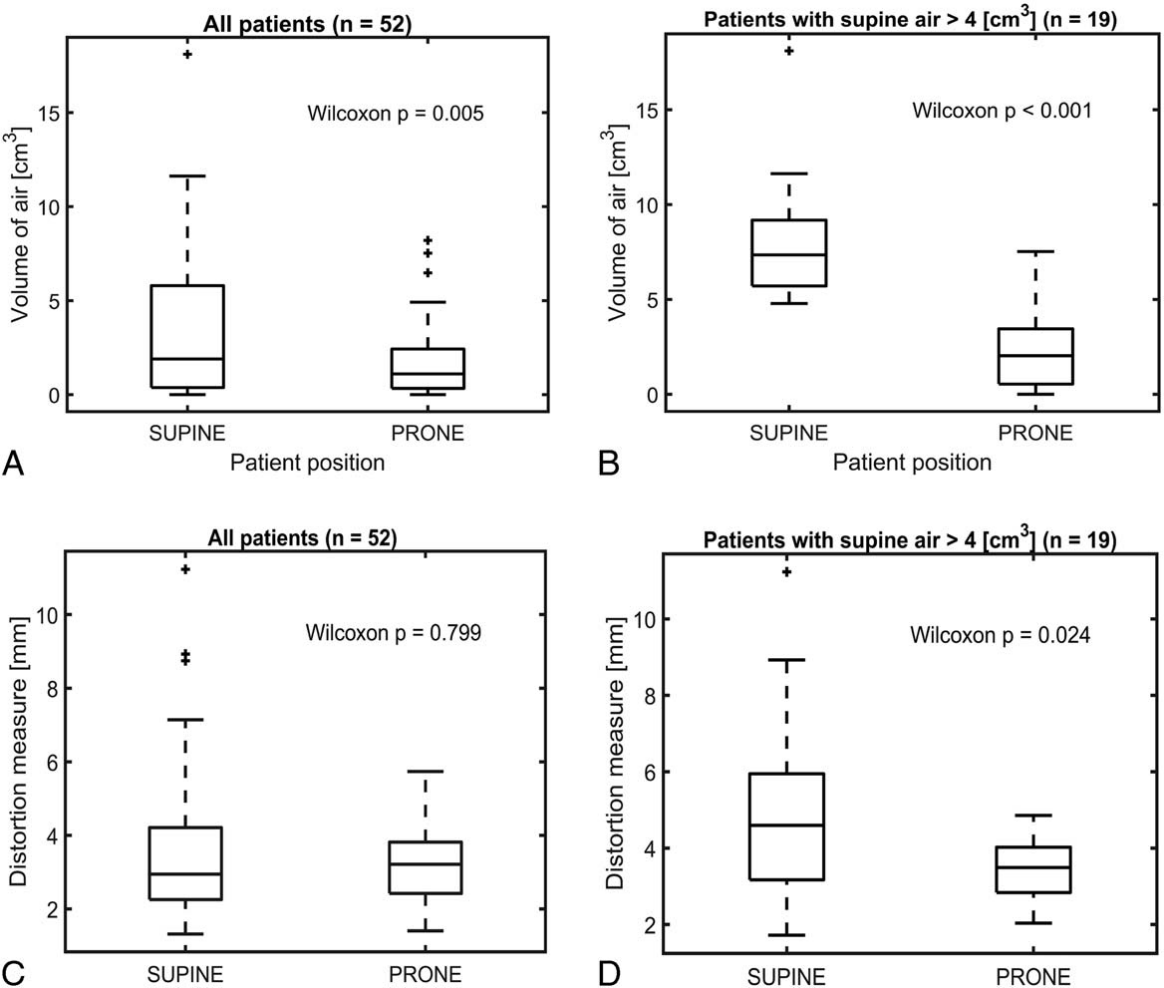
Box plots comparing patient prone and supine positioning. A and B, Volume of rectal air within 20 mm of the prostate. C and D, Distortion measurements for DW imaging of the posterior prostate. This is shown for all patients (A, C) and for the subset of patients with rectal air >4 cm^3^ on supine imaging (B, D). *P* values are shown for the statistical difference between the prone and supine results as evaluated using a paired Wilcoxon signed-rank test. DWI indicates diffusion-weighted imaging.

A scatterplot was generated showing the DWI distortion measure against rectal air volume within 20 mm of the prostate for all 52 patients, for both supine and prone cases (Fig. 6). Although Rectal Air_prox_ was shown to be significantly lower during prone positioning [median (IQR): 1.13 (0.34 to 2.43) cm^3^] compared with supine positions [1.96 (0.47 to 5.81) cm^3^] (*P* = 0.005) (Fig. 7A), there was no significant difference in Distortion_post_ between positions [prone: 3.21 (2.42 to 3.82) mm; supine: 2.95 (2.25 to 4.21) mm] (*P* = 0.80) (Fig. 7C).

Logistic regression yielded an air threshold value of V_thres-air_ = 4 cm^3^ (*P* = 0.008). Approximately 37% of patients (N = 19) had supine rectal air >4 cm^3^. Both Rectal Air_prox_ (*P* < 0.001) (Fig. 7B), and Distortion_post_ (*P* = 0.02) (Fig. 7D), were significantly reduced during prone imaging in this subgroup of patients [median (IQR): 2.03 (0.54 to 3.45) cm^3^ and 3.49 (2.84 to 4.03) mm, respectively] compared with supine positioning [7.35 (5.71 to 9.18) cm^3^ and 4.60 (3.17 to 5.95) mm, respectively].

### PI-RADS Categorization and Diagnostic Confidence

Compared with the standard-of-care protocol (Supine T2 + Supine DWI + Supine DCE; median: 4, IQR: 3 to 5), replacing supine DWI with prone DWI (Supine T2 + Prone DWI + Supine DCE; median: 4, IQR: 2 to 5) significantly increased the radiologist’s diagnostic confidence (*P* = 0.01; Supplemental Table S3, Supplemental Digital Content 2, http://links.lww.com/RLI/B68). The radiologist’s diagnostic confidence was significantly reduced when prone T2 was included compared with the standard-of-care protocol (*P* < 0.001; Supplemental Table S3, Supplemental Digital Content 2, http://links.lww.com/RLI/B68).

**FIGURE 8:**
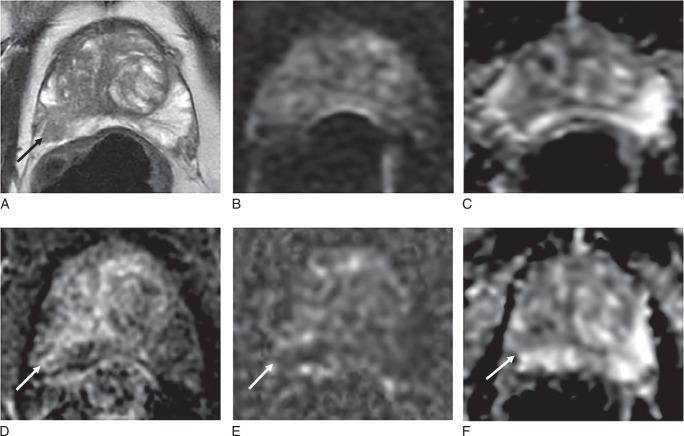
Example of a false-positive case with nondiagnostic supine DWI. Sixty-nine-year-old patient, PSA 5.17 ng/mL. A–D, Supine imaging shows ill-defined low T2 signal in the right mid PZ, PI-RADS 3 (arrow, A), poor quality DWI, PI-QUAL 1/4 (B, C), with focal early enhancement on DCE (arrow, D). E and F, Prone DWI is diagnostic and PI-RADS score 2. PI-RADS score 4 (3+1) using supine imaging alone with nondiagnostic DWI, but PI-RADS score 2 when using prone DWI.

**FIGURE 9:**
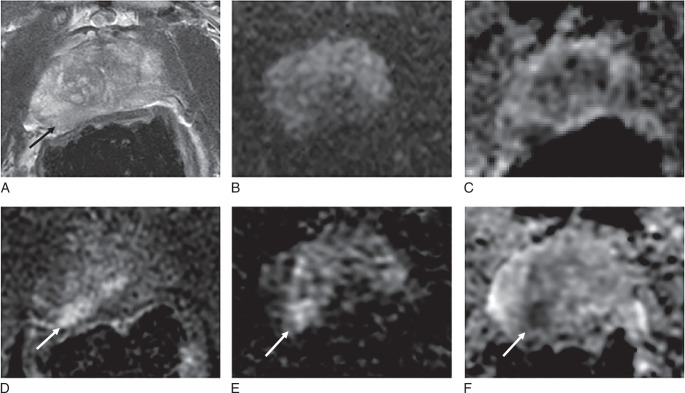
Example of a false-negative case with nondiagnostic supine DWI. Seventy-six-year-old patient, PSA 11.21 ng/mL. A–D, Supine imaging shows wedge-shaped mild low T2 signal in the right mid/apex PZ, PI-RADS 2 (arrow, A), poor quality DWI, PI-QUAL 1/4 (B, C), with marked focal early enhancement on DCE (arrow, D). E and F, Prone DWI is diagnostic and shows marked restricted diffusion, PI-RADS score 4. PI-RADS score 2 using supine imaging alone, with nondiagnostic DWI, but PI-RADS score 4 when using prone DWI. Targeted biopsy shows Gleason 3+4=7 in 1/3 cores with 5 mm max tumor length.

When comparing PI-RADS categories between these 2 protocols (Supplemental Table S4, Supplemental Digital Content 2, http://links.lww.com/RLI/B68), the overall distribution of PI-RADS categories was identical; however, one case was scored as PI-RADS 4 with the standard-of-care protocol but was scored as PI-RADS 2 with the alternative protocol (Fig. 8). Conversely, another case was scored as PI-RADS 2 with the standard-of-care protocol but as PI-RADS 4 with the alternative protocol (Fig. 9).

## DISCUSSION

This study investigated the impact of prone positioning on prostate MRI image quality in 52 patients. PI-QUAL v2 scores revealed a significant improvement in prone DWI image quality compared with supine DWI. Conversely, prone T2WI demonstrated significantly inferior image quality compared with supine T2WI due to motion artefacts. Quantitative analysis showed a significant decrease in rectal air volume adjacent to the prostate in the prone position. Notably, prone imaging significantly reduced quantitative DWI distortion in the posterior prostate region for patients with appreciable rectal air (≥4 cm^3^) on standard-of-care supine imaging. However, patients reported a general preference for supine positioning, and performing an additional prone DWI sequence routinely would add just over 8 minutes to a prostate MRI study.

High-quality prostate MRI is essential for optimal outcomes in the MRI-directed PCa diagnostic pathway.^4^ Suboptimal image quality can lead to increased uncertainty in MRI interpretation, increasing the risk of unnecessary biopsies due to false-negative MRI reports or underdiagnosis of clinically significant PCa due to false-positive reports.^4,5,8,16,17^ T2WI and DWI are the key sequences in mpMRI for the TZ and PZ, respectively, and are the only sequences included when a biparametric MRI approach is adopted.^18^ To standardize prostate MR image quality assessment, PI-QUAL v2 was applied by 2 experienced uroradiologists who were familiar with the scoring system, with inter-reader agreement found to be almost perfect across sequences in this study. These results are consistent with previous reports, which have reported moderate to almost perfect agreement levels.^19–22^

In this study, 42% (22/52) of supine DWI scans were rated as inadequate quality (PI-QUAL ≤ 2/4). Sackett et al^23^ reported 39% of DWI scans as inadequate, which is comparable to our findings.^23^ The EPI sequences used in DWI are highly sensitive to magnetic field inhomogeneity and vulnerable to the associated susceptibility artefacts, which are particularly pronounced at air-tissue interfaces. Ultimately, these inhomogeneities may result in image distortion and compromised image quality.^6,7,24^ Our study demonstrates that prone positioning significantly improves overall qualitative DW image quality and decreases the quantitative distortion in the posterior prostate region, particularly in cases with a clinically appreciable volume of rectal air at the rectoprostatic interface. This may explain why substituting supine DWI with prone DWI in the standard-of-care protocol significantly improved the radiologists’ diagnostic confidence. T2WI sequences, typically acquired using fast spin-echo (FSE) techniques, are usually less affected by susceptibility artefacts due to the application of a refocusing RF pulse between each line of k-space acquisition, mitigating the effects of field inhomogeneities.

Notably, prone T2W image quality was inferior to supine imaging due to the presence of motion artefact, and the radiologist’s diagnostic confidence reduced when prone T2WI was included. Consequently, a complete shift to a prone position may not improve overall quality, nor be tolerated by patients.^25^ Importantly, the differences in T2 quality were not simply due to reduced effects of hyoscine butylbromide, as significant motion artefacts were apparent in prone versus supine imaging, regardless of its administration. We postulate that prone imaging exacerbates transmission of respiratory motion to the abdomen and pelvis by splinting the chest wall. T2WI is more vulnerable to motion artefact, due to its longer acquisition time compared with other sequences,^6,7^ and the EPI sequence employed for DWI utilizes single-shot data collection, which captures all data in one rapid acquisition, in turn reducing vulnerability to motion artefacts.^6,7^ Indeed, 4 studies have reported increased patient motion and decreased tolerance during prone position scans.^25–28^ Wilder et al^25^ reported that 80% of patients preferred the supine position for scanning. In addition, Vargas et al^28^ used cine MRI to measure prostate motion in both supine and prone positions, demonstrating that prostate positional changes were significantly reduced in the supine position.

Routinely acquiring both supine and prone scans would increase scan time by around 8.5 minutes, potentially impacting clinical workflow efficiency. A more targeted approach is to selectively perform additional prone DWI for patients with appreciable rectal air, as this subgroup may benefit substantially from prone positioning. Although real-time estimation of rectal air volume—whether quantitative or qualitative—may be impractical in routine clinical settings, potentially limiting the direct translation of our findings into practice, on-table review has been reported for use of contrast^14^ and is advocated by the PI-RADS committee in this context.^29^

In addition, it has been reported that some prostate MRI lesions remain clearly visible even when image quality is deemed inadequate, making repeat or additional scans unnecessary.^5,17^ Similarly, if conspicuous lesions are present on supine images, an additional prone scan may not be needed, even in cases of significant rectal air and prominent DWI distortion.

Therefore, developing an automated tool for immediate use after a supine scan to detect substantial rectal air and facilitate prone image acquisition when clinically warranted could enhance imaging efficiency and quality. Moreover, if such a tool could predict the expected quality of supine DWI based on supine T2WI or even localizer images in real time, radiographers could make informed decisions about the optimal patient positioning for DWI. This would eliminate the need to routinely acquire supine DWI, reducing the added scan time of prone DWI to under 4 minutes, which is typically required for patient repositioning.

Our study has some limitations. First, images were acquired from a single 3T MRI machine at a single academic medical center, in a relatively small cohort. Future studies should investigate the generalizability of the effects of prone imaging in a multicenter setting with various MRI models. Second, PI-QUAL v2 was employed to evaluate image quality, which benefits from having dedicated 4-point T2WI and DWI quality scales; nevertheless, this remains a subjective assessment and has been shown to have only moderate inter-reader agreement.^11,19,22^ Third, only 37% of subjects had rectal air volumes exceeding the threshold for appreciable rectal air. While this is reflective of clinical practice and aligns with existing literature,^7^ a larger sample size with more patients exhibiting increased rectal air volumes could strengthen the study. Finally, for all patients, supine imaging was acquired first, and T2WI was always acquired before DWI; future work could address whether alternating this sequence has an independent impact on quality.

## CONCLUSIONS

In conclusion, qualitative analysis using PI-QUAL v2 demonstrated superior image quality for prone compared with supine DWI, particularly in patients with appreciable rectal air, while prone T2WI quality was inferior to supine T2WI. However, the majority of patients preferred supine positioning, and adding a prone scan increases scan time by ~8.5 minutes. Therefore, selectively acquiring prone scans for patients with significant rectal air could optimize image quality while minimizing patient discomfort and workflow disruption in MRI departments.

## Supplementary Material
