# Supplementary material for: Diffusion-weighted Imaging Distortion in Prostate MRI: A Cross-sectional Study Comparing Supine and Prone Positioning
Source: Invest Radiol. 2025 Nov 6;61(7):467–76. doi: 10.1097/RLI.0000000000001245 (PMC13232692; doi:10.1097/RLI.0000000000001245)
Supplement: Supplementary file 1 [file rli-61-467-s001.docx]

**Diffusion-weighted imaging distortion in prostate MRI: a cross-sectional study comparing supine and prone positioning**

**Supplementary figure:**


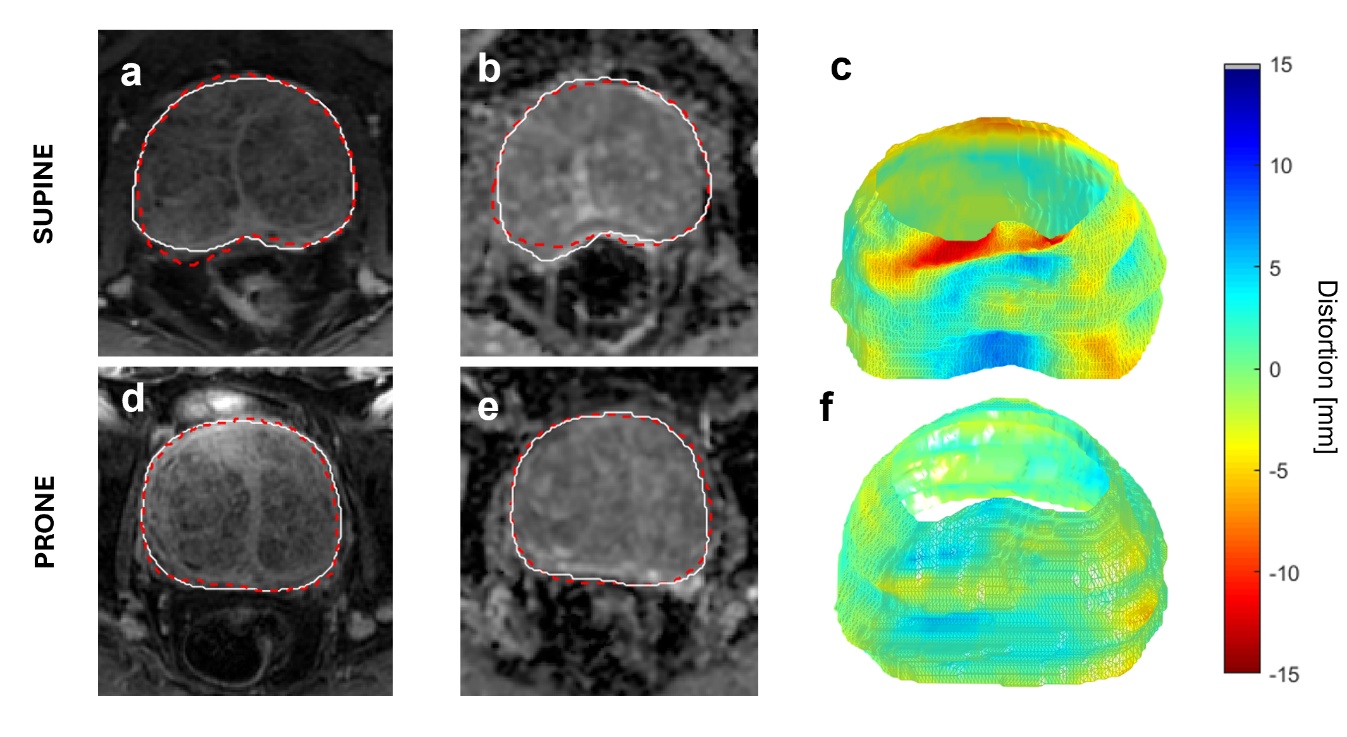


**Figure S1.** Sample images from one subject showing outlines on (a,d) reference images and (b,e) diffusion images, with 3-D renderings of the distortion between the two sets of outlines (c,f). Shown for patient positioned supine (top row) and prone (lower row).


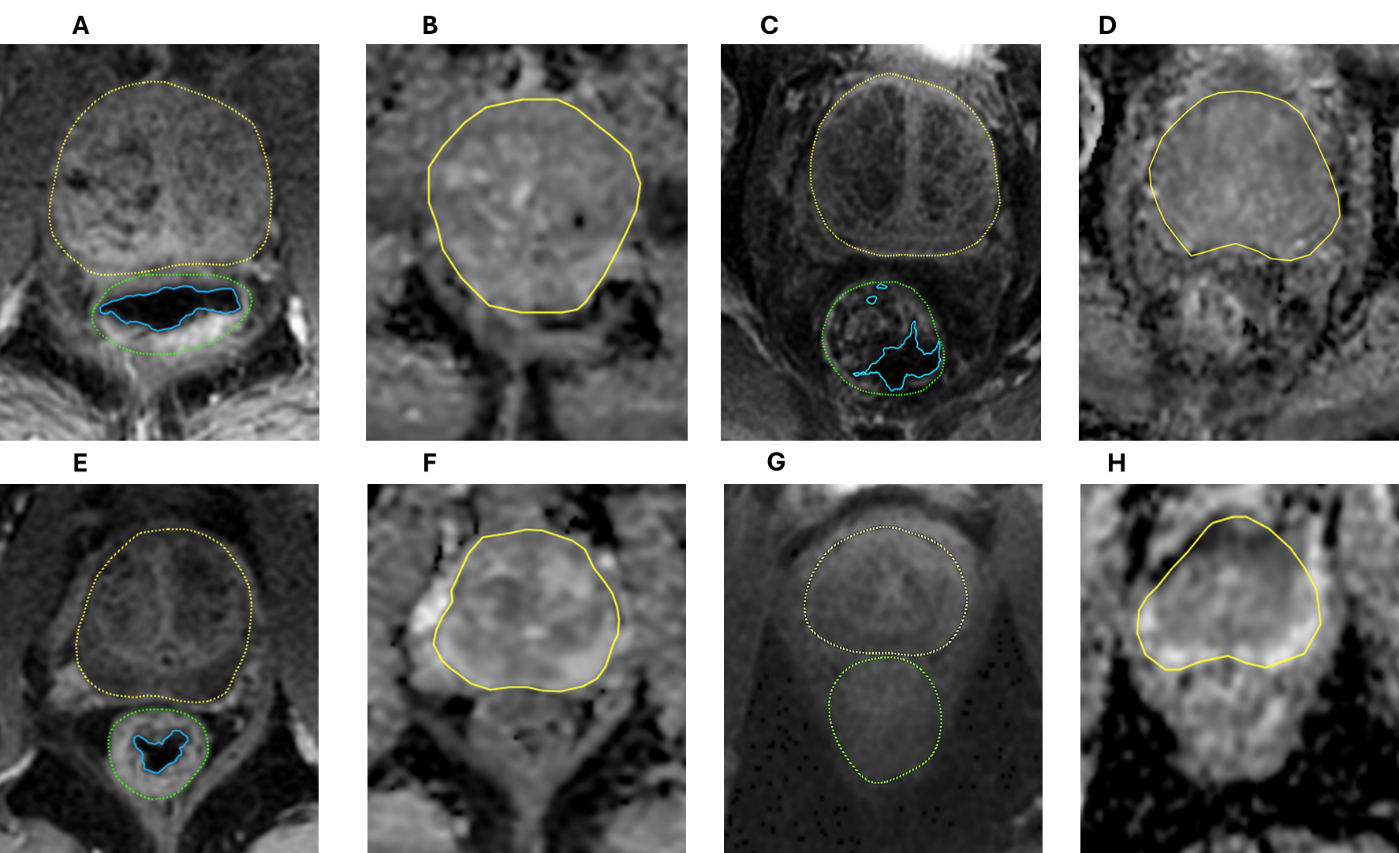


**Figure S2.** Imaging examples of a patient with an appreciable amount of rectal air posterior to the prostate gland in the supine position, shown on DCE (A), causing prominent distortion on the ADC map (B). When positioned prone, the rectal air moved away from the prostate gland, as seen on DCE (C), and distortion on the ADC map was reduced (D). In contrast, another patient without appreciable rectal air posterior to the prostate gland in the supine position showed minimal distortion on ADC maps in both supine and prone positions (DCE: E, G; ADC: F, H, respectively).

DCE = dynamic contrast-enhanced imaging; ADC = apparent diffusion coefficient
