# Supplementary material for: Diffusion-weighted Imaging Distortion in Prostate MRI: A Cross-sectional Study Comparing Supine and Prone Positioning
Source: Invest Radiol. 2025 Nov 6;61(7):467–76. doi: 10.1097/RLI.0000000000001245 (PMC13232692; doi:10.1097/RLI.0000000000001245)
Supplement: Supplementary file 2 [file rli-61-467-s002.docx]

**Diffusion-weighted imaging distortion in prostate MRI: a cross-sectional study comparing supine and prone positioning**

**Supplementary tables:**

| **Sequence** | **AC2 (95% CI)** |
| --- | --- |
| Supine DWI | 0.87 (0.80 – 0.94) |
| Prone DWI | 0.90 (0.83 – 0.98) |
| Supine T2WI | 0.87 (0.81 – 0.95) |
| Prone T2WI | 0.94 (0.88 – 1.00) |

**Supplemental Table S1**. Inter-Reader agreement on PI-QUAL scores attributed for each individual sequence.

AC2 = Gwet’s AC2 statistic, CI = confidence interval; DWI = axial diffusion-weighted imaging, T2WI = axial T2-weighted imaging

|  | T2WI | | DWI | |
| --- | --- | --- | --- | --- |
| Quality score | supine | prone | supine | prone |
| 4 | 24 | 1 | 18 | 34 |
| 3 | 21 | 0 | 12 | 10 |
| 2 | 4 | 9 | 7 | 7 |
| 1 | 3 | 31 | 8 | 1 |
| 0 | 0 | 11 | 7 | 0 |

**Supplemental Table S2**. Distribution of diagnostic PI-QUAL score for T2WI and DWI in supine and prone positions.

PI-QUAL = Prostate Imaging Quality, T2WI = T2-weighted imaging, DWI = diffusion weighted imaging.

| Diagnostic confidence | Supine T2 +  Supine DWI +  Supine DCE  **(****Standard-of-care)** | Supine T2 +  Prone DWI +  Supine DCE | Prone T2 + Prone DWI + Supine DCE | Prone T2 + Supine DWI + Supine DCE |
| --- | --- | --- | --- | --- |
| 1 | 0 | 0 | 0 | 3 |
| 2 | 2 | 0 | 1 | 37 |
| 3 | 13 | 2 | 36 | 10 |
| 4 | 23 | 30 | 13 | 2 |
| 5 | 14 | 20 | 2 | 0 |

**Supplemental Table S3.** Distribution of diagnostic confidence for supine T2 and supine DCE, combined with either supine DWI or prone DWI. Scoring ranged from “very unsure” (score 1) to “very sure” (score 5).

T2 = T2 weighted imaging, DWI = diffusion weighted imaging, DCE = dynamic contrast-enhanced imaging.

| PI-RADS | Supine T2 +  Supine DWI + Supine DCE  **(Standard-of-care)** | Supine T2 + Prone DWI + Supine DCE | Prone T2 + Prone DWI + Supine DCE | Prone T2 + Supine DWI + Supine DCE |
| --- | --- | --- | --- | --- |
| 1 | 6 | 6 | 2 | 1 |
| 2 | 27 | 27 | 31 | 32 |
| 3 | 4 | 4 | 2 | 2 |
| 4 | 8 | 8 | 9 | 9 |
| 5 | 7 | 7 | 8 | 8 |

**Supplemental Table S4.** PI-RADS distribution for different combinations of scan position protocols.

T2 = T2 weighted imaging, DWI = diffusion weighted imaging, DCE = dynamic contrast-enhanced imaging.
